# Effects of Climate Change Scenarios on Growth, Flowering Characteristics, and Honey Production Potential of *Pseudolysimachion rotundum* var. *subintegrum*

**DOI:** 10.3390/plants14111647

**Published:** 2025-05-28

**Authors:** Kyeong-Cheol Lee, Yeong-Geun Song, Hyun-Jung Koo, Kyung-Jun Kim, Hyung-Joo Kim, Ha-Young Baek, Sung-Joon Na

**Affiliations:** 1 Department of Crops and Forestry, Korea National University of Agriculture and Fisheries, Jeonju 54874, Republic of Korea; dlrud112@korea.kr (K.-C.L.); hjungkoo@korea.kr (H.-J.K.); 2 Department of Foresty, Jeonbuk National University, Jeonju 54896, Republic of Korea; songe2720@gmail.com; 3 Department of Environment and Forest Resources, College of Agriculture and Life Sciences, Chungnam National University, Daejeon 34134, Republic of Korea; touch93@cnu.ac.kr; 4 Department of Forest Sciences, Kongju National University, Yesan 32439, Republic of Korea; songbird57@naver.com; 5 Department of Bio-Functional Material, Kangwon National University, Samcheok 25913, Republic of Korea; hayoungbaek01@gmail.com; 6 Division of Special Forest Resources, National Institute of Forest Science, Suwon 16631, Republic of Korea

**Keywords:** *Pseudolysimachion rotundum* var. *subintegrum*, climate change, nectar secretion, honey production, photosynthesis, beekeeping

## Abstract

Climate change significantly influences plants’ physiology, flowering phenology, and nectar production, affecting pollinator interactions and apicultural sustainability. This study examines the physiological responses of *Pseudolysimachion rotundum* (Nakai) Holub var. *subintegrum* (Nakai) T.Yamaz. (Plantaginaceae) under projected climate change scenarios, focusing on flowering traits, nectar secretion, and honey production potential. Elevated CO_2_ levels enhanced its net photosynthesis and water-use efficiency, supporting sustained carbohydrate assimilation and promoting aboveground biomass accumulation. However, the increased nitrogen demand for vegetative growth and inflorescence production may have led to reduced allocation of nitrogen to the nectar, contributing to a decline in its amino acid concentrations. The flowering period advanced with rising temperatures, with peak bloom occurring up to four days earlier under the SSP5 conditions. While the nectar secretion per flower remained stable, an increase in floral abundance led to a 3.8-fold rise in the estimated honey production per hectare. The analysis of the nectar’s composition revealed that sucrose hydrolysis intensified under higher temperatures, shifting the nectar toward a hexose-rich profile. Although nectar quality slightly declined due to reductions in sucrose and nitrogen-rich amino acids, phenylalanine—the most preferred amino acid by honeybees—remained dominant across all scenarios. These findings confirm the strong climate resilience of *P. rotundum* var. *subintegrum*, highlighting its potential as a sustainable nectar source in future apicultural landscapes. Given the crucial role of nitrogen in both plant growth and nectar composition, future research should explore soil nitrogen dynamics and plant nitrogen metabolism to ensure long-term sustainability in plant–pollinator interactions and apicultural practices.

## 1. Introduction

Since the Industrial Revolution, anthropogenic emissions of greenhouse gases have gradually increased, leading to the global crisis of climate change. Climate change disrupts Earth’s previously stable energy balance due to rising atmospheric concentrations of carbon dioxide (CO_2_). This disruption results in extreme climate events such as global warming, droughts, and intense precipitation [1]. In response, the Intergovernmental Panel on Climate Change (IPCC) has developed Shared Socioeconomic Pathway (SSP) climate change scenarios to assess the future impacts of climate change and provide proactive information to minimize potential damage [2].

The Korea Meteorological Administration (KMA) published national projections based on the IPCC’s Sixth Assessment Report [3]. This report classifies future periods into the early (2021–2040), mid (2041–2060), and late (2081–2100) stages compared to the current climate (the 20-year average from 1995 to 2014) and presents the changes in environmental factors according to the climate change scenarios. According to SSP-based climate predictions, the global average temperature by the end of the 21st century is projected to be approximately 1.9 °C to 5.2 °C higher than that at present. When limited to the Northern Hemisphere, including the Korean Peninsula, the late-period temperature increases are expected to be +1.9 °C, +3.0 °C, +4.3 °C, and +5.2 °C under SSP1-2.6, SSP2-4.5, SSP3-7.0, and SSP5-8.5, respectively [4,5,6].

A rapid increase in atmospheric CO_2_ concentrations and temperatures exerts diverse effects on plant growth, leading to changes in their photosynthetic capacity, secondary metabolites, and bioactive compounds [6,7]. In particular, flowering in plants responds to environmental signals such as light and temperature. Consequently, climate change can alter the timing and duration of flowering, nectar production, and the chemical composition of nectar, which may have profound implications for reproductive success in both plant and animal species [1].

The vulnerability and sensitivity to climate change vary among plant species [6,7,8,9,10]. In the case of nectar plants, their role as a food resource for pollinators is crucial not only for the beekeeping industry but also for the ecosystem [11]. Therefore, it is essential to predict and prepare for changes in nectar plant productivity due to climate change.

To support the development of the beekeeping industry and increase beekeepers’ incomes, South Korea implemented the ‘Beekeeping Industry Promotion and Support Act’ in 2020. Efforts have been made to explore and expand various nectar plant species and develop technologies to enhance beekeeping productivity [12]. The number of domestic beekeeping farms increased significantly from 19,000 in 2011 to 29,000 in 2020, with the number of managed bee colonies rising from 1.76 million to 2.68 million over the same period, reflecting an annual growth rate of 5.3%. Despite this expansion, the average annual natural honey production dropped drastically from 25,000 tons in 2007–2011 to 13,000 tons in 2016–2020 [12]. This decline in honey production has been attributed to the heavy dependence (over 70%) on *Robinia pseudoacacia* L. (Fabaceae) as a single nectar source, along with unfavorable climatic conditions such as frequent rainfall and abnormal temperatures during the honey production season [13]. Consequently, there is growing interest in identifying nectar resources that exhibit high adaptability to climate change and have the potential to reshape the future beekeeping industry.

*Pseudolysimachion rotundum* var. *subintegrum*, commonly known as mountain speedwell, is a perennial herb belonging to the family Scrophulariaceae. It is a native and endemic species that thrives in grasslands in mountainous regions. The plant blooms from July to August, producing pale purple racemes 10–20 cm in length on its branches and stems. Due to its aesthetic appeal, it has been recognized as an ornamental plant and is also used in the treatment of asthma [14]. Notably, mountain speedwell flowers during the summer when nectar-producing plants are scarce and exhibits abundant flowering. Recent studies suggest that this plant can yield approximately 152 kg of honey per hectare, which is more than twice that of *Brassica napus* L. (Brassicaceae), making it a promising nectar resource [12].

Most studies on climate change and nectar-producing plants have primarily focused on food crops or alpine plants vulnerable to warming, often addressing ecological interactions with honeybees [1,6,15,16]. However, for the effective identification of superior nectar resources for the beekeeping industry, it is necessary to comprehensively examine physiological responses such as photosynthesis under climate change conditions and their impact on flowering time, flower abundance, honey yield, and nectar quality. Although studies using artificially controlled environments, such as growth chambers, have limitations in fully capturing all aspects of climate change responses, they are valuable for directly comparing the effects of elevated atmospheric CO_2_ and temperature, which are the most fundamental factors in climate change. Such approaches have been widely employed by researchers to elucidate basic plant traits and propose further studies [6,8,9,10]. Understanding plant development under elevated CO_2_ conditions is particularly important for predicting various adaptation responses to climate change [15].

This study investigates the physiological responses of *Pseudolysimachion rotundum* var. *subintegrum* under climate change scenario conditions, focusing on photosynthesis and water-use efficiency. Furthermore, we aim to analyze how these physiological changes affect its flowering time, flower abundance, honey production, and nectar quality. Through this study, we seek to confirm the value of mountain speedwell as a nectar resource and propose key indicators for selecting nectar plants with high adaptability to climate change. Additionally, we intend to evaluate the potential of nectar resources to expand the beekeeping industry and contribute to developing strategies for climate change adaptation.

## 2. Results

### 2.1. Photosynthesis and Stomatal Responses

**Figure 1:**
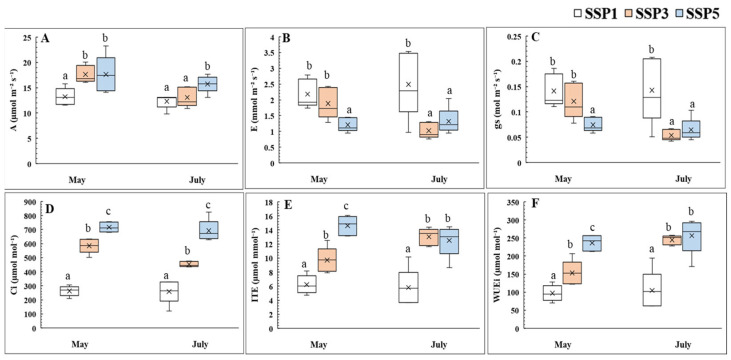
Box plots showing physiological traits of *P. rotundum* var. *subintegrum* measured in May and July under three climate scenarios (SSP1, SSP3, SSP5). (**A**) Net photosynthetic rate(A), (**B**) stomatal transpiration rate (E), (**C**) stomatal conductance (gs), (**D**) intercellular CO_2_ concentration (Ci), (**E**) instantaneous transpiration efficiency (ITE), and (**F**) intrinsic water-use efficiency (WUEi). Measurements reflect the plant’s photosynthetic response and water regulation under elevated CO₂ and temperature. Different letters indicate statistically significant differences among treatments based on Tukey’s HSD test (*p* < 0.05).

The photosynthetic and stomatal responses of *P. rotundum* var. *subintegrum* under different climate change scenarios are presented in Figure 1. Across all treatments, the intercellular CO_2_ concentration (Ci) remained slightly below the supplied atmospheric CO_2_ level but increased significantly with the severity of the scenario (*p* < 0.001). The net photosynthetic rate (A) was lowest in SSP1 in both May and July, while SSP5 showed an approximately 1.3-fold higher A compared to that in SSP1 (*p <* 0.05). SSP3 matched SSP5 in May but decreased later, aligning more closely with SSP1.

The stomatal transpiration rate (E) and stomatal conductance (gs) were significantly lower in SSP5 as early as May, decreasing to 52.8% of the values under SSP1 (*p* < 0.05). SSP3 remained similar to SSP1 in May but dropped by July, reaching values comparable to those in SSP5. In contrast, the instantaneous transpiration efficiency (ITE) and intrinsic water-use efficiency (WUEi) significantly increased with higher climate change scenario levels, with SSP3 and SSP5 showing 2.2–2.4 times higher values compared to those for SSP1 in July (*p <* 0.001).

### 2.2. Analysis of the Chlorophyll Content

In May, chlorophyll a, chlorophyll b, total chlorophyll, and the total chlorophyll/carotenoid ratio in SSP3 were slightly lower than those in SSP1 and SSP5, though these differences were not substantial (Table 1). By July, after four months of growth, a gradual decline in chlorophyll and carotenoid contents was observed in SSP3 and SSP5, where CO_2_ and temperature were elevated. Notably, chlorophyll a and b and the total chlorophyll content decreased significantly in SSP3 and SSP5 compared to these values in SSP1, reaching 78.0–78.8% and 57.8–59.6% of the SSP1 levels, respectively (*p <* 0.001). This decrease was more pronounced than that in the carotenoid content (82.9% and 28.6%, respectively).

The chlorophyll a/b ratio did not show significant differences across treatments (*p >* 0.05). However, the total chlorophyll/carotenoid ratio declined with a higher severity of the climate change scenario in July, primarily due to a sharper decrease in the chlorophyll content.

### 2.3. Chlorophyll Fluorescence Responses

**Figure 2:**
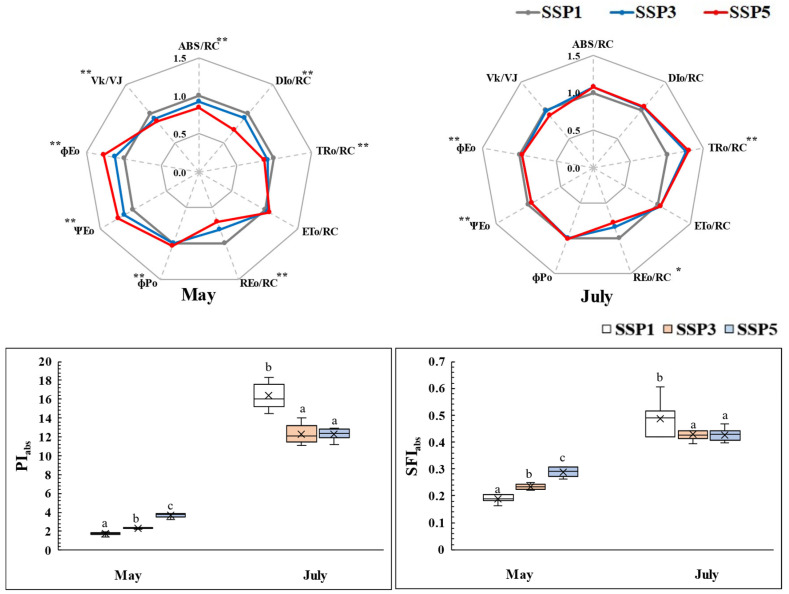
Spider plots comparing chlorophyll fluorescence parameters and photosynthetic vitality indices of *P. rotundum* var. *subintegrum* under three SSP scenarios in May and July. Parameters include energy absorption (ABS/RC), dissipation (DIo/RC), electron transport (ET0/RC, REo/RC), trapping efficiency (TRo/RC), and quantum yields (ΦPo, ΦEo, ΨEo). Higher values indicate stronger photosynthetic activity. Data are normalized to SSP1 for comparison. Asterisks and letters indicate significant differences (Tukey’s HSD: * *p* < 0.05, ** *p* < 0.001).

Monthly comparisons of the chlorophyll fluorescence response indices under different climate change scenarios (Figure 2) showed that in May, at the early growth stage, the energy flow per reaction center (ABS/RC, DIo/RC, TRo/RC, REo/RC) followed the trend SSP5 < SSP3 < SSP1 (*p <* 0.001). However, after more than four months of growth in July, REo/RC retained the same trend as that in May, whereas the other indices exhibited no significant differences (*p >* 0.05). Notably, the trend for TRo/RC was reversed, showing SSP1 < SSP5 = SSP3 (*p <* 0.001).

ΦPO, ΦEO, and ΨEO, which indicate the energy transfer efficiency and the fluorescence yield at different stages of photochemical reactions [17], followed the trend SSP1 < SSP3 < SSP5 in May (*p <* 0.05). However, by July, no significant differences were observed. Additionally, the variable fluorescence index V_K_/V_J_ was slightly lower in SSP5 compared to those in the other treatments in both May and July, though this difference was not statistically significant in July (*p >* 0.05).

The photosynthetic performance indices PI_ABS_ and SFI_ABS_ increased significantly from May to July, with PI_ABS_ increasing 9.4-, 5.3-, and 3.3-fold in SSP1, SSP3, and SSP5, respectively. SFI_ABS_ increased 2.6-, 1.8-, and 1.5-fold, respectively, indicating that PI_ABS_ responded more sensitively. Due to these variations, the trend in the climate change scenario differed between months, with SSP5 exhibiting the highest values in May (*p <* 0.001), whereas SSP1 showed the highest values in July (*p <* 0.001).

### 2.4. Growth and Flowering Characteristics

The differences in the biomass accumulation at the end of the growing season (late October) under different climate change scenarios are presented in Table 2. As the severity of the climate change scenario increased, the total biomass also increased, with SSP5 exhibiting the highest biomass accumulation (*p <* 0.001). Although there were no statistically significant differences in the belowground biomass (*p >* 0.05), the aboveground biomass increased by 20.2% in SSP3 and 36.9% in SSP5 compared to that in SSP1, which was further confirmed by the differences in the shoot-to-root (S/R) ratio (*p <* 0.001). These results indicate that the observed increase in total biomass under the climate change conditions was primarily driven by an increase in aboveground biomass. A comparison of the inflorescence length and width (Table 2) revealed that the inflorescence length was 1.5 times longer in SSP3 (30.4 ± 1.8 cm) and 1.2 times longer in SSP5 compared to that in SSP1. In contrast, the inflorescence width ranged from 1.5 to 1.8 cm, with no statistically significant differences among treatments (*p >* 0.05).

The number of inflorescences per plant progressively increased under more severe climate change scenarios, with 50.1 ± 30.3 in SSP1, 72.2 ± 31.3 in SSP3, and 89.0 ± 47.4 in SSP5. Similarly, the number of flowers per inflorescence followed the same trend, increasing from 205.0 ± 63.2 (SSP1) to 264.1 ± 50.6 (SSP3) and 347.4 ± 86.8 (SSP5). These findings indicate that as the severity of the climate change scenario increased, the number of inflorescences and flowers per inflorescence increased up to 1.8-fold and 1.7-fold, respectively.

**Figure 3:**
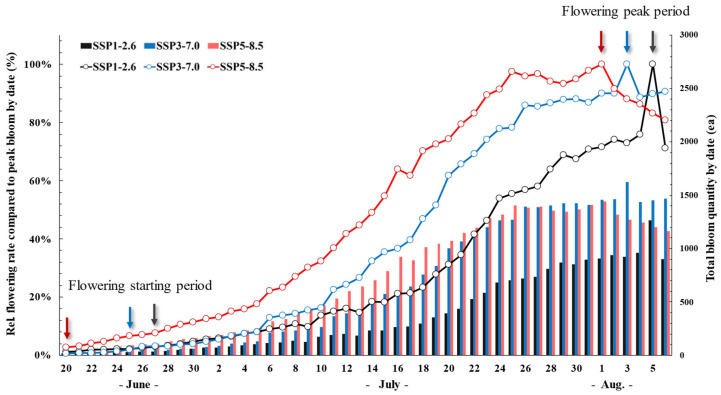
Temporal changes in the relative flowering rate (line graph) and the cumulative bloom quantity (bar graph) of *P. rotundum* var. *subintegrum* under three climate change scenarios (SSP1, SSP3, SSP5) from late June to early August. Flowering onset occurred earlier under higher-emission scenarios, with SSP5 initiating on 21 June compared to 28 June under SSP1. The peak flowering dates were also advanced by up to 4 days under SSP5. These shifts suggest that elevated CO_2_ and temperature accelerate floral development and flowering synchrony.

To examine the effect of climate change on flowering timing, the flowering start date was defined as the point when 3% of the peak flowering population had bloomed. The flowering start date of *P. rotundum* var. *subintegrum* occurred earlier under more extreme climate change conditions, with SSP3 and SSP5 initiating flowering on 26 June and 21 June, respectively, compared to 28 June in SSP1 (+2 and +7 days earlier). Similarly, the peak flowering dates were observed on 5 August, 3 August, and 1 August in SSP1, SSP3, and SSP5, respectively, meaning that peak flowering was reached 2 and 4 days earlier in SSP3 and SSP5 compared to SSP1 (Figure 3).

### 2.5. The Abundance of Nectar

The nectar secretion per flower at different flowering stages—the beginning-bloom stage (BBS), the mid-bloom stage (MBS), and the end-bloom stage (EBS)—showed a significant increase from the first to the second day in all treatments except SSP5 at the BBS (*p >* 0.05) (Table 3) (*p <* 0.05).

At the BBS, the nectar secretion in SSP5 was 0.26 μL/flower on the first day, approximately 1.8 times higher than that in SSP1 and SSP3 (*p <* 0.001), though no significant differences were observed on day 2. At the MBS, no differences were detected among treatments on either day. However, at the EBS, the nectar secretion in SSP5 was 0.09 μL/flower on day 1 and 0.24 μL/flower on day 2, significantly lower values than those for SSP1, by 42.2% and 59.9%, respectively (*p <* 0.05).

### 2.6. Floral Nectar Content and Composition

**Figure 4:**
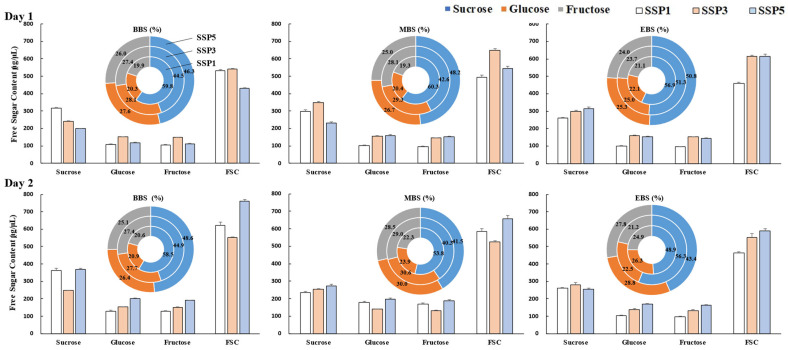
Free sugar content (bar graphs) and sugar composition (donut charts) of nectar from *P. rotundum* var. *subintegrum* across three flowering stages—BBS (beginning-bloom stage), MBS (mid-bloom stage), and EBS (end-bloom stage)—on days 1 and 2 of anthesis under three SSP scenarios. Each bar represents the sucrose, glucose, fructose, and total free sugar content (FSC) per unit volume (μg/μL). SSP5 generally exhibited a higher FSC on day 2, especially at the BBS (up to 762.0 μg/μL). The circular graphs show the percentage contributions of each type of sugar to the total FSC, indicating a shift toward more balanced glucose/fructose levels under elevated CO_2_.

The sucrose, glucose, fructose, and total soluble sugar contents per unit volume are presented in Figure 4 and Table 4. In *P. rotundum* var. *subintegrum*, significant differences were observed in response to climate change, flowering stage, and their interactive effects (*p <* 0.001). In the early flowering stage (BBS), both SSP1 and SSP5 showed an increase in the sucrose, glucose, fructose, and FSC levels on day 2 compared to those on day 1. Notably, SSP5 exhibited a substantial increase in the FSC from 431.1 μg/μL to 762.0 μg/μL, approximately 1.8 times higher. In contrast, SSP3 showed minimal variations between days 1 and 2 across all sugar types.

During the MBS, SSP1 and SSP5 maintained elevated sugar contents on day 2 compared to those on day 1, whereas SSP3 exhibited no significant change, reflecting a trend similar to that in the early flowering stage.

In the late flowering stage (EBS), SSP1 exhibited a significant increase on day 2 compared to day 1, similar to the patterns observed in the early- and full-bloom stages. Conversely, SSP5 displayed increased glucose, fructose, and total soluble sugars on day 2, but the sucrose content decreased by approximately 8.6%, reaching 255.9 μg/μL. Unlike the previous stages, SSP3 in the late flowering stage exhibited higher sucrose, glucose, fructose, and total sugar contents on day 1 than those on day 2, by 20.6%, 46.8%, 48.0%, and 32.3%, respectively.

When examining the sugar dynamics across flowering stages, SSP1 showed a gradual decline in sucrose levels from early to late flowering on both days 1 and 2. Glucose and fructose remained stable on day 1 but increased slightly on day 2. As a result, the total sugar content on day 2 remained relatively unchanged, while on day 1, an 86.3% reduction in the FSC occurred compared to that in the early flowering stage. In SSP3, no notable differences were observed between the early and full bloom stages, but in the late flowering stage, the sucrose content on day 1 increased by 30.3% (314.6 μg/μL) compared to that in the early stage, making this the peak period for FSC accumulation. However, while the sucrose levels slightly increased on day 2, the glucose and fructose levels declined sharply, resulting in the FSC reaching only 84.2% of that in the early flowering stage.

In SSP5, the sucrose content on day 1 gradually increased as flowering progressed, whereas on day 2, a marked decline was observed, indicating an inverse trend. The FSC also increased on day 1 but decreased on day 2. While the glucose and fructose levels remained stable on day 1, a substantial reduction occurred on day 2 in the late flowering stage, significantly contributing to the decline in the FSC.

### 2.7. The Amino Acid Content and Composition

The highest amino acid content was observed on day 1 under SSP3-BBS, reaching 4419.8 ± 83.1 mg/L, followed by SSP1-MBS, with 4430.3 ± 187.1 mg/L, while the lowest was found in SSP5-MBS at 1476.6 ± 72.2 mg/L, as shown in Table 5. Similarly, on day 2, SSP1-MBS exhibited the highest content of 4335.0 ± 157.7 mg/L, whereas SSP5-MBS recorded the lowest value of 1198.5 ± 89.5 mg/L. In general, the MBS treatments tended to show higher total amino acid contents than those in the BBS and EBS, particularly under the SSP1 and SSP3 scenarios.

A total of 17 amino acids were detected in *P. rotundum* var. *subintegrum*, showing notable compositional variations depending on the flowering stages and climate change scenarios. Notably, during full bloom, SSP5 exhibited the most distinct amino acid composition among the treatments (Table 5). In the early flowering stage, except for day 1 of SSP3, essential amino acids accounted for 66.0–78.6% of the total amino acid content, being 2.0–3.7 times higher than non-essential amino acids.

**Figure 5:**
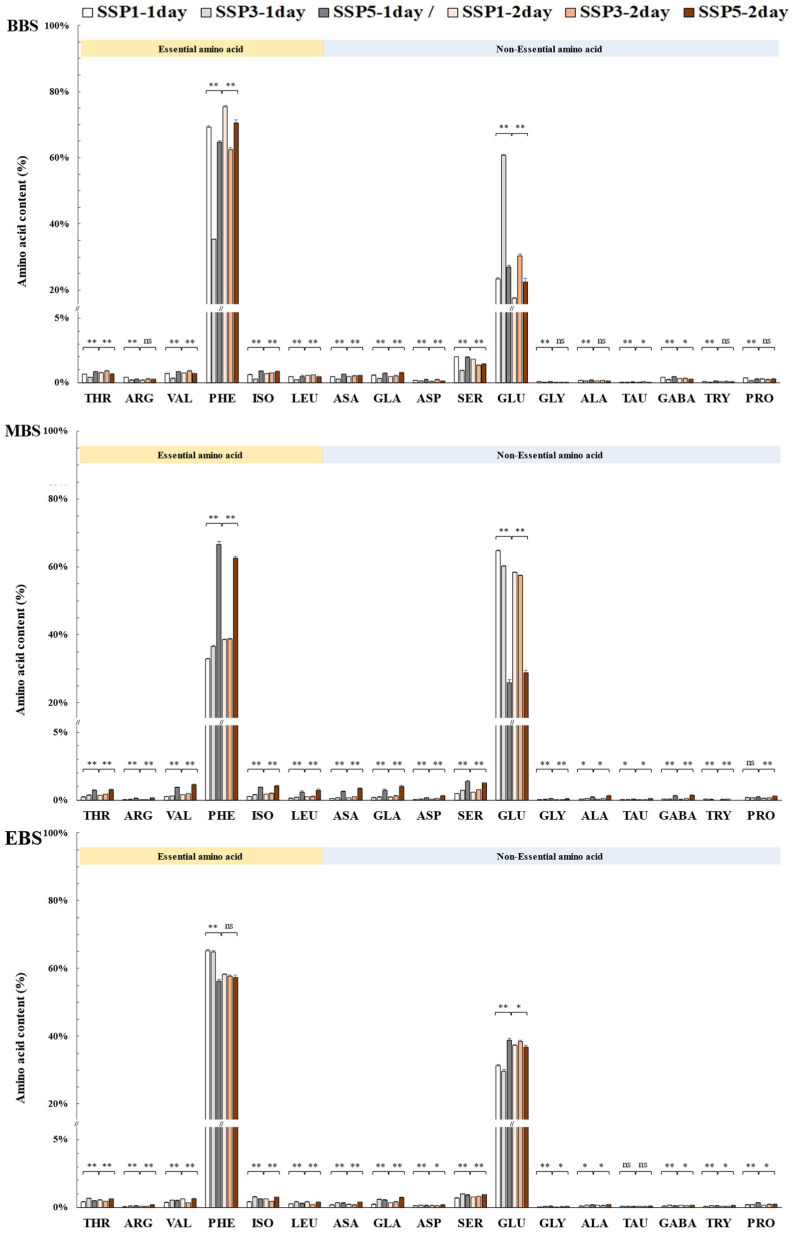
Amino acid composition of nectar from *P. rotundum* var. *subintegrum* across three flowering stages—the beginning-bloom stage (BBS), mid-bloom stage (MBS), and end-bloom stage (EBS)—and two sampling days under three SSP scenarios. Essential amino acids (left) and non-essential amino acids (right) are shown as percentages of the total amino acid content. Phenylalanine (PHE) and glutamine (GLU) were the most dominant amino acids, with marked compositional shifts observed under the SSP5 scenario. Asterisks indicate statistically significant differences (* *p* < 0.05, ** *p* < 0.001); ns = not significant. Amino acid abbreviations: THR (threonine), ARG (arginine), VAL (valine), PHE (phenylalanine), ISO (isoleucine), LEU (leucine), ASA (aspartic acid), GLA (glutamic acid), ASP (asparagine), SER (serine), GLU (glutamine), GLY (glycine), ALA (alanine), TAU (taurine), TRY (tyrosine), and PRO (proline).

Phenylalanine consistently represented the highest proportion (62.5–75.5%) of total amino acids across most treatments. However, glutamine was exceptionally elevated under SSP3 on day 1 (244.3 mg/L), accounting for 60.8% of the total amino acid content, which was 4.8–6.5 times higher than that in the other treatments. Other essential amino acids, including threonine (0.7–0.9%), valine (0.7–0.9%), isoleucine (0.6–0.9%), leucine (0.5–0.6%), and arginine (0.2–0.4%), were present in relatively low amounts. Non-essential amino acids mainly consisted of glutamine (17.5–30.3%), serine (1.4–2.0%), aspartic acid (0.5–0.7%), glutamic acid (0.5–0.8%), proline (0.2–0.4%), asparagine (0.1–0.3%), GABA (0.3–0.53%), and alanine (0.2%). Glycine, taurine, and tyrosine were detected at trace levels, each accounting for approximately 0.1% (Figure 5).

During full bloom (MBS), phenylalanine and glutamine remained the dominant amino acids. Notably, except for SSP5 (66.4–70.1% essential amino acids), most treatments exhibited lower proportions of essential amino acids (33.8–40.5%) than those of non-essential amino acids. As the severity of the climate change scenarios increased, the phenylalanine content increased while the glutamine content decreased. On day 1, the proportions of phenylalanine were 33.8%, 38.0%, and 70.1% under SSP1, SSP3, and SSP5, respectively, whereas the proportions of glutamine were 66.2%, 60.0%, and 29.9%. On day 2, the proportions of phenylalanine increased to 40.2%, 40.5%, and 66.4%, with the proportions of glutamine slightly decreasing to 59.8%, 59.5%, and 33.6%, respectively.

In the late flowering stage (EBS), phenylalanine (58.3–66.8%) and glutamine (33.2–41.7%) remained the predominant amino acids, with essential amino acids consistently exceeding non-essential amino acids by 1.4–2.1 times across all treatments. In SSP5, the phenylalanine levels on day 1 were slightly lower (58.3%), with higher glutamine levels (41.7%), but their composition had nearly stabilized on day 2. Although the same 17 amino acids were consistently detected across all flowering stages, their compositional ratios exhibited slight variations (Figure 5).

### 2.8. Estimation of Honey Production

Assuming that honeybees’ foraging behavior is random across all flowering stages, the average nectar secretion per flower on day 2 of anthesis and its free sugar content were used to estimate the FSC per flower (Table 6). The results showed that SSP1 had a slightly higher FSC per flower (0.22 µg/flower); however, this difference was not statistically significant (*p >* 0.05).

In contrast, the estimated honey production per plant showed a clear increasing trend as the severity of climate change increased, with values of 2.6 g/plant, 3.6 g/plant, and 7.1 g/plant under SSP1, SSP3, and SSP5, respectively. A similar trend was observed for the estimated honey yield per hectare. Considering a standard planting density for herbaceous plants (spacing of 30 × 30 cm, corresponding to 110,000 plants per hectare), the estimated honey production under SSP3 (401.1 kg/ha) was 1.2 times higher than that under SSP1, while under SSP5 (772.8 kg/ha), it was 2.7 times higher than that under SSP1.

To provide an integrated overview of the physiological and reproductive responses of *P. rotundum* var. *subintegrum* under varying climate change scenarios, a comparative summary table was prepared (Table 7). This table consolidates the major findings on the photosynthetic traits, biomass accumulation, flowering patterns, and nectar quantity and composition, as well as amino acid content, across the SSP1, SSP3, and SSP5 scenarios. By summarizing the diverse measurements reported in Section 2.1, Section 2.2, Section 2.3, Section 2.4, Section 2.5, Section 2.6, Section 2.7 and Section 2.8, this synthesis facilitates a clearer understanding of how *P. rotundum* var. *subintegrum* adapts to elevated CO_2_ and temperature conditions. In particular, it highlights the trade-offs between increased floral productivity and potential declines in nectar quality under more severe climate scenarios, thereby offering practical implications for pollination services and future apicultural resource planning.

## 3. Discussion

### 3.1. Photosynthesis and Stomatal Response

Nectar is produced through the accumulation of photosynthetically derived carbohydrates in the parenchyma cells near the nectary, which are subsequently secreted along with water [18,19,20]. Therefore, the net photosynthetic rate directly or indirectly contributes to nectar secretion.

Atmospheric CO_2_ enters the plant through the stomata and diffuses into the chloroplast stroma, where carboxylation occurs. Elevated atmospheric CO_2_ levels enhance the CO_2_ diffusion pressure, increasing the CO_2_ saturation within the intercellular spaces and temporarily boosting photosynthesis through the carbon fertilization effect [21,22,23,24]. This enhanced photosynthesis increases the concentration of total non-structural carbohydrates (sugars and starch) within leaf tissues [25,26,27]. The present study confirmed that as the climate change scenarios intensified, the intercellular CO_2_ concentration (Ci) in *P. rotundum* var. *subintegrum* also increased, leading to the saturation of available CO_2_ within the plant, which ultimately resulted in an overall rise in its net photosynthesis.

However, the ability to sustain this enhancement throughout the later growth stages varies by species. Many plants exhibit an initial increase in their net photosynthesis under elevated CO_2_ but experience photosynthetic suppression over extended growth periods [10,24]. For instance, in the forest medicinal plant Angelica gigas, the net photosynthesis in May under the SSP5 scenario was the highest among treatments but declined sharply by August [10]. Likewise, *Cnidium officinale* Makino (Apiaceae) showed a pronounced decline in net photosynthesis by July after an initial increase in spring [28]. This phenomenon is attributed to starch accumulation and thickening of the cell walls under prolonged high-CO_2_ conditions, thereby increasing the CO_2_ diffusion resistance within the chloroplasts and gradually impairing the photosynthetic capacity [24,29].

However, *P. rotundum* var. *subintegrum* maintained a high net photosynthesis even until July, the peak flowering period under the elevated climate change scenarios. This suggests that the accumulation and utilization of photosynthetic assimilates could contribute to increased nectar production.

In general, angiosperms exhibit reduced stomatal conductance and transpiration under atmospheric CO_2_ concentrations exceeding 600 µmol·m^−2^·s^−1^ due to osmotic stress [10,24,30]. *P. rotundum* var. *subintegrum* also exhibited a decreased stomatal conductance and transpiration rate, which increased its intrinsic water-use efficiency (ITE, WUEi), indicating a regulatory mechanism for controlling water loss during photosynthesis [24]. This increased water availability plays a crucial role in reproductive responses to climate change [31]. Moreover, the abundant sugars produced via photosynthesis act as osmoprotectants, stabilizing the cell membranes and maintaining turgor pressure, thereby enhancing the tolerance to abiotic stress and sustaining flowering under climate change conditions [32].

In conclusion, elevated climate change scenarios enhanced photosynthetic activity, supplying the carbohydrates essential for growth and flowering. Simultaneously, the water consumption increased, prompting adaptive responses for efficient water regulation.

### 3.2. Analysis of the Chlorophyll Content

Chlorophyll and carotenoids are photosynthetic pigments closely related to leaves’ nitrogen content and photosynthetic capacity [33,34]. Under the SSP1 scenario, which closely resembled the current atmospheric environment, *P. rotundum* var. *subintegrum* exhibited a typical developmental pattern, with increasing chlorophyll and carotenoid contents from early spring growth through to summer flowering to optimize light energy harvesting for photosynthesis. SSP3 showed a similar trend.

However, under SSP5, which involved the highest increases in CO_2_ concentrations and temperature, the chlorophyll and carotenoid contents decreased significantly from May to July, reaching distinctly lower levels compared to those in SSP1. This change is likely due to accelerated nitrogen depletion from the soil due to the rapid growth facilitated by high photosynthesis [26]. Furthermore, under strong summer sunlight combined with elevated temperatures, delayed chloroplast formation serves as a protective mechanism against photodamage [35]. Although environmental stress generally causes chloroplast degradation and early leaf senescence, leading to reduced pigment contents [10,35], *P. rotundum* var. *subintegrum* maintained nearly stable pigment levels from early growth until July. This suggests an adaptive resource allocation strategy focused on the stability of the photosynthetic apparatus rather than promoting premature leaf senescence under climate change conditions.

### 3.3. Analysis of Chlorophyll Fluorescence

OJIP fluorescence analyses quantitatively evaluate the physiological responses to environmental stress before visible damage symptoms appear, serving as an early indicator of stress tolerance [17,23,35].

Plants sensitive to high temperatures, strong light, and drought typically exhibit increased ABS/RC and DIo/RC, reducing the absorbed light energy per reaction center and dissipating excess energy as heat to stabilize the photosynthetic apparatus [10,29]. However, *P. rotundum* var. *subintegrum* exhibited no significant differences in ABS/RC and DIo/RC under SSP3 and SSP5 compared to that in SSP1, indicating that this excess energy did not severely damage the photosynthetic apparatus.

The maximum quantum yield of primary photochemistry (ΦPO), as well as the electron transport efficiency (ΦEO, ΨEO), exhibited significantly favorable trends under the higher climate change scenarios in May (*p* < 0.001) and remained stable through July. The ratio of the variable fluorescence at K and J steps (V_K_/V_J_), an indicator of inactivation of the donor side of photosystem II under stress [35,36], decreased under SSP5 compared to SSP1. This suggests that even under elevated CO_2_ and temperature conditions, *P. rotundum* var. *subintegrum* maintains a stable electron transport process in photosystem II, demonstrating strong adaptation to climate change stress.

The photosynthetic vitality indices, such as PI_ABS_ and SFI_ABS_, reflect the structural and functional performance of the photosynthetic apparatus and are sensitive indicators of environmental stress [17,24,37]. Many plants exhibit significant declines in PI_ABS_ and SFI_ABS_ after prolonged growth under climate change conditions, indicating stress effects [9,10,28]. However, *P. rotundum* var. *subintegrum* maintained high photosynthetic vitality under these conditions, particularly increasing PI_ABS_ and SFI_ABS_ values in July to support the energy demands of flowering. The highest energy conservation efficiency in May under SSP5 (*p <* 0.001) likely reflects early leaf maturation and favorable CO_2_ and temperature conditions. By July, SSP5 and SSP3 exhibited slight declines in the vitality indices compared to those in SSP1 (*p <* 0.05), reflecting increased temperature stress and water demands. Nevertheless, the overall photosynthetic vitality remained high across treatments, confirming *P. rotundum* var. *subintegrum*’s strong adaptation to climate change.

Thus, *P. rotundum* var. *subintegrum* exhibited enhanced energy conservation efficiency in photosystem II during the flowering stage, supported by a structurally and functionally resilient photosynthetic apparatus, maintaining stability even under elevated CO_2_ and temperature conditions, confirming its high adaptability to climate change.

### 3.4. Growth and Flowering Characteristics

Long-term cultivation under climate change conditions exhibited a progressive increase in the overall plant growth. Generally, elevated atmospheric CO_2_ levels enhance plant growth, particularly promoting the accumulation of shoot biomass in herbaceous plants, as reported in previous studies [10,38,39]. Flowering characteristics, including flowering time and quantity, are also known to accelerate with increased CO_2_ levels as a result of enhanced photosynthetic rates [40]. While the underground biomass of *P. rotundum* var. *subintegrum* did not show significant differences under climate change conditions (*p >* 0.05), its aboveground biomass progressively increased, resulting in a 14.3% and 21.3% greater total biomass in the SSP3 and SSP5 scenarios, respectively, compared to that in SSP1 (*p <* 0.001).

Moreover, flowering characteristics such as the inflorescence length and width, number of inflorescences per plant, and number of flowers per inflorescence increased significantly with elevated climate change scenario levels, except for inflorescence width (*p <* 0.05), mirroring the trend observed for aboveground biomass accumulation. Early-spring flowering plants generally require vernalization, wherein exposure to low winter temperatures promotes flowering. In such cases, winter warming could paradoxically reduce flowering [40]. However, *P. rotundum* var. *subintegrum* is a day-neutral plant with no vernalization requirements [41], and its summer flowering suggests that temperature and CO_2_ conditions directly influence the flowering traits throughout its growth period. Notably, *P. rotundum* var. *subintegrum* has an indeterminate inflorescence, in which flowers continue to develop at the apex while flowering progresses from the lower part of the inflorescence [42]. Consequently, inflorescence length can be highly responsive to the growth conditions during the flowering period.

These findings indicate that under climate change conditions, sustained enhancement in the net photosynthetic rate (Figure 1) before and during flowering led to the increased accumulation of photosynthates, which were preferentially allocated to the aboveground growth. This, in turn, promoted the production of the reproductive organs, such as the numbers of inflorescences and flowers per inflorescence. Therefore, climate change conditions significantly impact not only vegetative growth but also the development of reproductive structures in *P. rotundum* var. *subintegrum*.

Changes in flowering time due to rising atmospheric CO_2_ and temperature levels are among the most pronounced phenological responses in plants, with earlier flowering being the most common trend [15,43,44]. This early flowering response is attributed to the accelerated attainment of a critical biomass threshold required for flowering due to enhanced photosynthesis under elevated CO_2_ [45], as well as increased sucrose transport from the leaves to the shoot apical meristems under higher temperatures [46]. In *P. rotundum* var. *subintegrum*, the onset of flowering advanced by up to seven days under climate change conditions, and peak flowering occurred up to four days earlier.

In conclusion, *P. rotundum* var. *subintegrum* exhibited an overall advancement in flowering time and significant increases in both its vegetative and reproductive growth under elevated temperature and CO_2_ conditions. These results suggest that climate change may enhance the potential of *P. rotundum* var. *subintegrum* as a nectar source plant for pollinators. These findings support the idea that photosynthetic vitality indices (PI_ABS_ and SFI_ABS_ etc.) and external morphological characteristics (inflorescence length, number of inflorescences per plant, etc.) are potentially useful for assessing physiological stress and are key indicators for selecting nectar plants with high adaptability to climate change.

### 3.5. Nectar Characteristics

The timing and pattern of nectar secretion vary among plant species [47], and nectar production may decrease or cease over time [48,49]. In addition, nectar secretion and sugar concentrations within the same species can be influenced by flowering stage and time of day [50]. Under climate change scenarios, the nectar volume per flower in *P. rotundum* var. *subintegrum* increased significantly on day 2 compared to that on day 1 (*p <* 0.05). This trend has also been observed in other nectariferous plants such as *Chaenomeles speciosa* Nakai (Rosaceae) [49] and *Prunus × yedoensis* Matsum. ex Koidz. (Rosaceae) [51], suggesting that the increased nectar secretion on day 2 resulted from the accumulation of initially secreted nectar as the flower matured.

Cultivation under climate change conditions resulted in nectar volumes ranging from 0.09 to 0.26 μL/flower on day 1 and 0.24 to 0.41 μL/flower on day 2, displaying treatment-dependent variations. Compared to field-grown *P. rotundum* var. *subintegrum* reported by [12] (0.07 μL/flower on day 1, 0.30 μL/flower on day 2), the nectar secretion was slightly higher under climate change conditions. However, compared to major woody nectariferous plants such as *R. pseudoacacia* (2.2 μL/flower), *Castanea crenata* Siebold & Zucc. (Fagaceae) (26.8 μL/catkin), *Hovenia dulcis* Thunb. (Rhamnaceae) (4.2 μL/flower), and *Camellia japonica* L. (Theaceae) (3.6–118.5 μL/flower) [52,53,54], *P. rotundum* var. *subintegrum* exhibited a markedly lower nectar production per flower. This is likely due to its small flower size and elongated, indeterminate inflorescence structure.

The nectar secretion trends varied depending on the flowering stage and climate change scenario. Under SSP5 conditions, nectar secretion peaked during the early flowering stage but significantly decreased towards the late flowering stage, particularly showing a notable reduction on day 1 of anthesis. In contrast, under SSP1 conditions, the nectar secretion tended to increase slightly during the late flowering stage. These results suggest that SSP5 promotes nectar secretion primarily in the early flowering phase, whereas SSP1 exhibits higher nectar production during the late flowering phase.

Nectar secretion is strongly influenced by environmental factors such as temperature and relative humidity, as well as selective reabsorption by the plant [55]. Low relative humidity generally leads to increased nectar concentrations due to water evaporation, while temperature affects photosynthesis, thereby influencing nectar production. Lower temperatures typically reduce nectar secretion [56,57]. The variations in nectar secretion across flowering stages in *P. rotundum* var. *subintegrum* under climate change conditions are likely linked to environmental factors and photosynthetic activity. Specifically, the elevated temperature and CO_2_ conditions in SSP5 appear to have promoted nectar secretion immediately after anthesis. However, this effect was not sustained throughout the flowering period, suggesting a regulatory mechanism to maintain nectar homeostasis and optimize energy utilization. According to [58], plants continuously regulate nectar concentrations in response to environmental changes, often involving cycles of nectar secretion, cessation, and reabsorption during flowering [59]. The observed trends in *P. rotundum* var. *subintegrum* suggest that climate change conditions may result in a temporal concentration of nectar secretion during the early flowering stages. From an ecological perspective, this may imply a shorter time frame for pollinators to access nectar as an energy resource.

### 3.6. Changes in Nectar’s Sugar Composition

The pathway of nectar secretion involves modified stomatal structures distributed in the nectary region, with nectar precursors synthesized via the Calvin cycle and transported through phloem sap. During this process, sugars are temporarily stored and hydrolyzed in the parenchyma cells within the nectary, ultimately leading to nectar secretion [18,60].

Nectar consists of approximately 80% water and contains dissolved sugars, amino acids, organic acids, proteins, lipids, vitamins, and minerals [18]. Consequently, fluctuations in nectar’s water content significantly impact the concentration of these constituents per unit volume. The water content of nectar varies widely depending on the environmental conditions, which in turn influences nectar’s volume and concentration. Specifically, under hot and dry conditions, the nectar secretion decreases due to water loss, whereas the concentration of free sugars increases correspondingly [12]. Similarly, in *P. rotundum* var. *subintegrum*, the free sugar content per unit volume (FSC) exhibited characteristic trends. On the day of anthesis (day 1), SSP5 exhibited the lowest FSC among all treatments during the early flowering stage. However, the FSC gradually increased, eventually exceeding the levels observed in SSP1. This trend strongly correlated with the gradual decrease in the nectar secretion per flower in SSP5 as anthesis progressed. Similar tendencies were observed under different climate change conditions and on day 2 of flowering. However, not all variations in the FSC were solely due to differences in nectar secretion volume. A comparison between day 1 and day 2 of anthesis revealed a significant increase in the nectar secretion volume on day 2, regardless of flowering stage or climate conditions, yet the FSC did not decrease; rather, it increased. This suggests that the variation in the free sugar concentrations cannot be fully explained by nectar secretion volume alone.

It is well established that the brief surge in nectar secretion at anthesis is primarily regulated by the hydrolysis of pre-stored starch granules rather than the immediate supply of sucrose from photosynthesis [61,62,63]. Ref. [56] demonstrated through defoliation experiments that only a fraction of daily nectar secretion relies on daily photosynthesis, with a significant portion being mobilized from stored assimilates. Thus, the high FSC observed on day 2 in SSP5 under climate change conditions can be attributed to the hydrolysis of carbohydrates accumulated through relatively active photosynthesis before and during anthesis (Figure 1). This indicates that the secretion of free sugar in *P. rotundum* var. *subintegrum*’s nectar is strongly influenced by the overall photosynthetic activity throughout its growth period.

Furthermore, the analysis of the changes in the free sugar content across flowering stages under different climate change scenarios revealed a compensatory relationship between the sugar concentrations on days 1 and 2 of anthesis. Specifically, in SSP5, the FSC was the lowest on day 1 compared to that in SSP1 and SSP3 but increased substantially on day 2, surpassing all other conditions. This pattern was generally consistent across all conditions, resulting in an average FSC range of 536–596 µL/µg across all treatments over the two days. These findings suggest that the free sugar levels in nectar are regulated not only by concurrent photosynthesis but also by the mobilization of stored carbohydrates [18]. Plants actively maintain nectar concentration homeostasis in response to environmental fluctuations [58].

The fluctuation in the free sugar content in *P. rotundum* var. *subintegrum* indicates that the low FSC on day 1 is balanced by intensive hydrolysis of stored carbohydrates on day 2. This phenomenon benefits pollinators such as honeybees by ensuring higher sugar concentrations on day 2, compensating for the relatively low yield and energy acquisition from flowers visited on day 1. Additionally, since *P. rotundum* var. *subintegrum* continuously produces new inflorescences post-anthesis, with varying flowering stages and floral retention periods per inflorescence, it can attract pollinators to a greater number of flowers. As a result, honeybees can efficiently gather energy from multiple sources, while *P. rotundum* var. *subintegrum* enhances its chances of pollination, demonstrating an optimal mutualistic efficiency.

However, the role of the free sugar balance in nectar in response to climate change and other environmental factors has broad ecological implications. Therefore, the results of this study should be interpreted with caution, and further research with continuous observations is necessary to gain deeper insights.

In general, the free sugars in nectar primarily consist of sucrose, fructose, and glucose. In *P. rotundum* var. *subintegrum*, sucrose was the dominant component under all conditions, indicating that sucrose content is a key determinant of variations in the FSC. The sucrose-to-hexose (S/H) ratio is a critical factor influencing pollinator attraction [64,65]. High-sucrose nectar is preferred by long-tongued pollinators such as honeybees, whereas hexose-rich nectar attracts short-tongued pollinators such as flies [66,67].

Ref. [66] classified nectar based on the S/H ratio into four categories: sucrose-dominant (a ratio > 1.0), sucrose-rich (0.5–1.0), hexose-rich (0.1–0.5), and hexose-dominant (a ratio < 0.1). *P. rotundum* var. *subintegrum* remained in the sucrose-dominant group (with a S/H ratio > 1.0) on both days 1 and 2 in the SSP1 conditions, while in SSP3 and SSP5, it mostly fell into the sucrose-rich group, except for in late-stage SSP3, in which it shifted into the sucrose-dominant category (Table 2 and Table 3).

The slight decrease in the S/H ratio in SSP3 and SSP5 during the early and peak flowering stages suggests that higher temperatures under climate change conditions led to increased sucrose hydrolysis, with more sucrose converted into fructose and glucose [68]. Indeed, the resulting fructose-to-glucose ratio remained close to 1:1, supporting this hypothesis.

Ultimately, *P. rotundum* var. *subintegrum* exhibited temporary variations in the sugar concentrations of its nectar depending on the flowering stage and climate conditions. However, it maintained nectar homeostasis by synthesizing additional free sugars from stored carbohydrates. While the nectar’s sugar composition was generally suitable for honeybee attraction, increased sucrose hydrolysis under the climate change conditions during the early and peak flowering stages slightly reduced nectar quality. These results suggest that while the nectar volume increased under certain conditions, the decline in sucrose concentrations may have reduced the nectar’s palatability and potentially influenced pollinator visitation.

### 3.7. Changes in Amino Acid Composition

Amino acids play a crucial role in determining the taste and nutritional value of floral nectar, which in turn influences pollinator visitation [69]. The composition of amino acids varies among plant species, and even within the same species, differences in both type and quantity have been observed [12].

In this study, the *P. rotundum* var. *subintegrum* nectar consistently contained six essential and eleven non-essential amino acids across all treatments. However, the total free amino acid content markedly declined under the climate change scenarios, particularly during the mid-blooming stage (MBS). This reduction was most pronounced under the SSP5 scenario, where the amino acid concentrations were significantly lower compared to those in the present climate (SSP1), suggesting that future climate conditions may negatively affect nectar quality.

Interestingly, elevated temperature and CO_2_ stimulated nectar production, thereby increasing the nectar yield per plant [70]. However, this enhancement was accompanied by a sharp decline in the amino acid concentrations per unit volume. This inverse relationship between nectar quantity and quality is likely driven by a dilution effect, potentially exacerbated by a metabolic imbalance in carbon-to-nitrogen assimilation [71]. Enhanced photosynthesis and biomass accumulation under elevated CO_2_ and temperature conditions likely accelerate the nitrogen uptake from the soil, leading to local depletion and reduced allocation to nectar amino acid biosynthesis [72]. This effect was particularly evident during the mid-bloom stage (MBS), when the floral density and biomass peaked, intensifying amino acid dilution. The impact was further exacerbated under the high-emission SSP5 scenario, where stronger CO_2_ fertilization effects and increased nitrogen demand likely amplified the observed reduction in nectar quality.

Ref. [73] identified ten essential amino acids that honeybees cannot synthesize internally or convert from other amino acids, necessitating their intake through nectar and pollen. He reported that essential amino acids should constitute approximately 1–4% of the total amino acid content. In *P. rotundum* var. *subintegrum*, phenylalanine was the most abundant essential amino acid, while glutamine was the most abundant non-essential amino acid. This high phenylalanine content holds ecological significance, as this amino acid is a potent phagostimulant that attracts pollinators [12,74]. Similarly, *Dendropanax morbiferus* H.Lév. (Araliaceae)’s nectar was reported to contain a high proportion (67.2%) of phenylalanine [75].

The composition of amino acids in nectar also has significant implications for pollinators’ physiology and behavior. Glutamine, the most abundant non-essential amino acid in *P. rotundum* var. *subintegrum* nectar, has been reported at high levels in *C. japonica* (29.0%) and *D. morbifera* (11.0%) [67,75]. This amino acid is a key component in purine nucleotide synthesis and serves as a precursor for neurotransmitters such as serotonin [12]. Additionally, taurine is presumed to play a role in the development of flight muscles in winged insects, while proline is an essential energy source for long-distance bee flights and contributes to innate immunity in bees [12,76,77,78,79]. Glutamic acid has been reported to reduce oxidative stress in both larvae and adult *R. pseudoacacia* [80,81]. Furthermore, gamma-aminobutyric acid (GABA) acts synergistically with taurine to suppress excessive excitatory states under stress conditions and is commonly found in Mediterranean plant species, significantly influencing the foraging preferences of honeybees and megachilid bees [82].

Crucially, numerous studies have demonstrated a direct positive relationship between the nitrogen availability in the soil and nectar amino acid concentrations, with higher nitrogen levels facilitating amino acid biosynthesis and accumulation in nectar [83,84]. Thus, the observed decrease in nectar amino acids is not solely a result of passive dilution but also reflects nitrogen limitations, which actively constrain amino acid biosynthesis. In such conditions, nitrogen is prioritized for structural and reproductive growth at the expense of secondary functions like nectar amino acid production.

The inverse relationship between nectar quantity and quality under climate change scenarios has profound implications for ecological interactions and apicultural practices [6,85,86,87]. While increased nectar yields may boost the honey production per hive, the concurrent decline in amino acid concentrations could compromise honey’s nutritional quality, potentially affecting bees’ metabolism and immune function and overall colony resilience. Furthermore, amino acids contribute not only to honey’s nutritional value but also to its organoleptic properties, posing an additional challenge for sustainable beekeeping in future climates.

Given the strong link between nectar amino acid synthesis and plants’ nitrogen status, adopting soil nitrogen management strategies—such as fertilization or soil amendments—could offer a practical approach to mitigating these negative impacts [83,84,88]. Enhancing nitrogen availability in the soil could support the amino acid concentrations in nectar while maintaining floral abundance and nectar volume. Nevertheless, further research is essential to establish the optimal management practices that balance enhanced plant productivity with the preservation of nectar’s nutritional quality.

In conclusion, *P. rotundum* var. *subintegrum* displayed considerable resilience to future climate conditions by maintaining its nectar production and retaining key attractant amino acids like phenylalanine. However, the pronounced decline in its total amino acid concentrations—particularly under extreme scenarios like SSP5—suggests that climate change could impair nectar quality and consequently affect pollination services and honey quality. Although the total nectar production and floral abundance increased, concurrent reductions in sucrose and amino acid concentrations may limit the attractiveness and nutritional value of nectar to pollinators. This trade-off between nectar quantity and quality warrants careful ecological consideration, as qualitative changes in nectar composition could influence pollinator behavior, including visitation frequency and foraging efficiency. To ensure long-term ecological and apicultural sustainability, future research should explore how such shifts in nectar traits affect pollinator–plant interactions under natural field conditions and variable resource environments.

### 3.8. Estimation of Honey Production

Nectar production is primarily influenced by plants’ physiological responses to environmental factors, especially temperature and CO_2_ levels [6,69]. In general, moderate warming may enhance nectar secretion, while excessive heat reduces both nectar quantity and secretion rates [50,89,90]. However, the degree of vulnerability varies among species depending on their ecological adaptations [6].

In this study, *P. rotundum* var. *subintegrum*, an open grassland species, maintained stable nectar production per flower under all climate change scenarios, even under severe warming conditions (in the SSP5 scenario). These results suggest that this species exhibits a high degree of resilience to environmental stress, similar to other sun-adapted Mediterranean plants [6]. Interestingly, elevated temperatures and increased atmospheric CO_2_ did not reduce the nectar output per flower, and enhanced vegetative growth led to significant increases in the number of inflorescences and flowers per plant. Consequently, the total honey production per plant and per unit area increased up to 3.8-fold under climate change scenarios.

This increase in nectar’s availability may play a critical role in sustaining pollinator populations. A consistent nectar supply is essential for pollinator health, reproduction, and colony maintenance [87]. The abundance of high-quality nectar from *P. rotundum* var. *subintegrum* under future climate conditions indicates that this species may act as a consistent and reliable nectar resource for pollinators, buffering the negative impacts of the climate-induced floral resource decline observed in other ecosystems.

From a practical perspective, the substantial increase in the total nectar yield is expected to benefit apiculture, potentially enhancing the honey production in mountainous regions where *P. rotundum* var. *subintegrum* is distributed. Furthermore, the stable nectar composition, characterized by a high proportion of essential amino acids and phenylalanine, may enhance honey’s quality and attractiveness to honeybees [74].

Overall, these findings suggest that *P. rotundum* var. *subintegrum* could function as a resilient floral resource under future climate conditions, providing stable nectar supplies for pollinators and supporting sustainable apiculture. Given the increasing concerns about declines in floral resources due to climate change, identifying species with such ecological stability is essential for both pollination services and agricultural productivity. This study highlights the potential of *P. rotundum* var. *subintegrum* as an important forage plant that may contribute to maintaining pollinator populations and honey production even under changing environmental conditions.

However, it is important to note that this study was conducted under controlled environment chamber conditions, which allow for the precise manipulation of temperature and atmospheric CO_2_. Such controlled settings may not fully reflect the complexity of natural ecosystems, including biotic interactions and seasonal variability. Therefore, further field-based studies are necessary to confirm the broader ecological applicability of these results.

## 4. Materials and Methods

### 4.1. TheClimate Change Scenario Setup

Two-year-old seedlings of *P. rotundum* var. *subintegrum* were obtained from the National Institute of Forest Science, the Republic of Korea. On 12 April 2024, the seedlings were transplanted into 2.5 L pots (16 cm in height) filled with a 1:1 (*v*/*v*) mixture of horticultural nursery media (Punong, Gyeongju, Republic of Korea) and decomposed granite. In early June, when the plants exhibited vigorous growth, they were transplanted into larger black pots with a capacity of 23 L and a height of 26 cm. The experiment was conducted from 12 April to 30 November 2024 in a climate-controlled environment within the Climate Change Education Center at Korea National University of Agriculture and Fisheries. Three SFDS chambers (Soil Fruit Daylit System chambers, PTW Freiburg, Freiburg, Germany) were used to precisely regulate the atmospheric CO_2_ concentrations, temperature, and humidity under natural light conditions.

**Figure 6:**
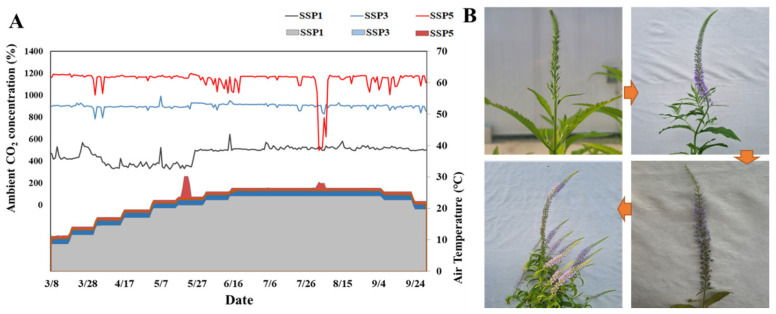
(**A**) Ambient CO_2_ concentration and air temperature profiles applied in the controlled environment chambers for each SSP scenario (SSP1, SSP3, SSP5) from March to September. SSP5 featured the highest and most prolonged CO_2_ and temperature elevations. (**B**) Representative images of the floral development stages of *P. rotundum* var. *subintegrum* under elevated climate conditions, including inflorescence elongation, early blooming, peak flowering, and post-flowering. These images illustrate the accelerated floral development observed under SSP5.

Each chamber contained 25 plants, with five replications per climate change scenario (SSP1-2.6: SSP1, SSP3-7.0: SSP3, and SSP5-8.5: SSP5), for a total of 75 experimental plants. The atmospheric CO_2_ concentrations for each scenario were set at 445 ppm, 872 ppm, and 1142 ppm, respectively, while the temperature treatments were based on the mean temperature in Jeonju over the past 20 years (1995–2014). The treatment conditions were established as +1.8 °C, +3.6 °C, and +4.4 °C above the baseline [4]. The microclimate within the chambers was monitored using environmental sensors (CO_2_: GMW86P, Vaisala, Vantaa, Finland; RH: HMW82, Vaisala, Vantaa, Finland; temperature: RBF185L, Pyromation, Inc., Fort Wayne, IN, USA). The mean measured CO_2_ concentrations during the experiment were 471 ppm, 902 ppm, and 1142 ppm for the SSP1, SSP3, and SSP5 treatments, respectively, while the daily mean temperatures were 20.3 °C, 22.1 °C, and 23.1 °C, closely aligning with the initially set conditions (Figure 6). To minimize temperature fluctuations that could affect the flowering phase, summer temperatures were maintained at the late-June levels from mid-June to late August.

The soil moisture content was monitored using a portable soil moisture meter (WT1000N, Mirae Sensor Co., Ltd., Seoul, Republic of Korea) every 1–3 days at approximately 10:00 a.m. in three replicates per chamber. The soil moisture levels were maintained between 4 and 5% before irrigation and 20 and 25% after irrigation. During the high-temperature period (July–August), each plant received an average of 4 L of water per irrigation.

### 4.2. Photosynthetic and Stomatal Responses

Photosynthetic and stomatal responses were measured in May and July using a portable photosynthesis system (Li-6800, Li-Cor Inc., Lincoln, NE, USA). Five replications per treatment were made, with the photosynthetic photon flux density (PPFD) set at 1200 µmol·m^−2^·s^−1^ using an LED light source attached to the measurement device. The net photosynthesis rate (A), stomatal transpiration rate (E), and stomatal conductance (gs) were recorded. The intrinsic water-use efficiency (WUEi) and the instantaneous transpiration efficiency (ITE) were calculated based on these parameters [24,35]. The airflow into the chamber was maintained at 600 μmol·s^−1^, and the temperature was held at 25 ± 1 °C during measurements (Figure 6).

### 4.3. Analysis of the Chlorophyll Pigment Content

The chlorophyll and carotenoid contents were analyzed in May and July, coinciding with the photosynthesis experiment. Five replications per treatment were conducted. Leaf samples (0.1 g) were extracted in 10 mL of dimethyl sulfoxide (DMSO) in 20 mL glass vials and incubated at 65 °C for six hours [91]. The absorbance at 663 nm, 645 nm, and 470 nm was measured using a UV/VIS spectrophotometer (HP 8453, Hewlett Packard, Wilmington, DE, USA) to calculate the chlorophyll a and b, total chlorophyll (a + b), and carotenoid contents [35]. These contents were calculated using the following equations:

- Chlorophyll a = 12.7 A663 − 2.69 A645;
- Chlorophyll b = 22.9 A645 − 4.68 A663;
- Total chlorophyll = 20.29 A645 + 8.02 A663;
- Total carotenoids = (1000 A470 − 1.82 Chl a − 85.02 Chl b)/198.

### 4.4. Analysis of the Chlorophyll Fluorescence

To compare the photosystem II activity and photosynthetic apparatus efficiency across climate change scenarios, chlorophyll fluorescence responses were assessed using a Plant Efficiency Analyzer (Hansatech Instrument Ltd., King’s Lynn, UK). The OKJIP analysis was conducted monthly from May to July, with five replications per treatment. The leaves were dark-adapted for 20 min before exposure to a 3500 µmol·m^−2^·s^−1^ light pulse for one second. The chlorophyll fluorescence intensities were recorded at 50 µs (O phase), 300 µs (K phase), 2 ms (J phase), 30 ms (I phase), and 500 ms (P phase), and the biophysical parameters were calculated (Table 8) [17,24,35].

### 4.5. Growth and Flowering Characteristics

The growth parameters were assessed on October 16 (the end of the flowering period). The shoots and roots were separated; dried at 80 °C for 48 h using a drying oven (DS-80-5, Dasol Scientific Co., Ltd., Gyeonggi-do, Republic of Korea); and weighed in triplicate to determine the dry mass and the S/R ratio. The flowering characteristics were assessed by counting the total number of inflorescences per plant. Additionally, the number of flowers per inflorescence and the length and width of the central inflorescence were measured in ten replicates per scenario.

### 4.6. Nectar Secretion Characteristics

The nectar secretion characteristics of *P. rotundum* var. *subintegrum*, which exhibits an indefinite inflorescence, were investigated by categorizing the flowering period into three stages—early, peak, and late—based on the progression of flowering within an inflorescence. For each stage, flowers were further classified into those on day 1 and day 2 of anthesis. In the early flowering stage, flowering initiated from the lower part of the inflorescence. The flowers that had bloomed within the first day were marked, and on the second day, nectar was collected from both the previously marked flowers (day 2) and the newly opened flowers (day 1). The peak flowering stage was defined as the point when more than 50% of the inflorescence had bloomed, while the late flowering stage was identified as when over 80% of the inflorescence had bloomed and flower abscission had begun at the basal part of the inflorescence. Nectar collection in the peak and late flowering stages followed the same method as that in the early stage. Flower collection was conducted between 4:00 p.m. and 6:00 p.m. [51], with four replicates for each climate change scenario (Figure 6). To collect nectar at each flowering stage, the petals were removed, and nectar was extracted using a microcentrifuge (Microfuge 16, Beckman Coulter, Indianapolis, IN, USA) at 3000 rpm for 4 min. The collected nectar was quantified using a 100 μL microliter syringe (Hamilton Company, Reno, NV, USA) and further purified through filtration using a 0.45 μm membrane filter (Whatman, UK). The purified nectar was diluted 10-fold with 80% ethanol, stored in Eppendorf vials, and preserved at −20 °C in an ultra-low-temperature freezer until the high-performance liquid chromatography (HPLC) analysis [12].

### 4.7. Free Sugar Analysis

The free sugar analysis was conducted using high-performance liquid chromatography (HPLC; Dionex Ultimate 3000, (Thermo Fisher Scientific, Waltham, MA, USA) with four replicates. Triple-distilled water was used as the mobile phase at a flow rate of 0.5 mL/min, and the column temperature was set to 80 °C. Detection was performed using an RI-101 detector (Shodex, Tokyo, Japan) with an Aminex 87P column (Bio-Rad Laboratories, Hercules, CA, USA). The quantification was based on an external standard method using integration values, with sucrose, glucose, fructose, and galactose (Sigma-Aldrich, St. Louis, MO, USA) as the reference standards.

### 4.8. Free Amino Acid Analysis

To analyze free amino acids, nectar samples were derivatized using O-phthalaldehyde (OPA) and fluorenylmethyl chloroformate (FMOC) and analyzed in four replicates. The sample was sequentially mixed with borate buffer, OPA/mercaptopropionic acid (MPA), and FMOC reagent, followed by an HPLC analysis using an Agilent 1200 series HPLC system (Agilent Technologies, Santa Clara, CA, USA). The mobile phases consisted of Solution A (10 mM Na_2_HPO_4_ and 10 mM Na_2_B_4_O_7_·10H_2_O, pH 8.2) and Solution B (water:acetonitrile:methanol = 10:45:45, *v*/*v*). The gradient conditions were set as follows: 100:0 (A:B, *v*/*v*) initially, transitioning to 55:45 at 26–28 min, 0:100 at 28–30.5 min, and returning to 100:0 from 30.5 min onward. The flow rate was 1.5 mL/min, with an injection volume of 1 mL, and the column temperature was maintained at 40 °C using an Inno column C18 (Innopiatech, Daejeon, Republic of Korea). Detection was performed using both UV and fluorescence detectors. The UV detector was set at 338 nm, while the OPA derivative was detected at an excitation wavelength of 340 nm and an emission wavelength of 450 nm. The FMOC derivative was detected at an excitation wavelength of 266 nm and an emission wavelength of 305 nm.

### 4.9. Estimation of Honey Production

The honey production per plant (g/plant) was estimated based on the nectar secretion volume (μL), the free sugar content per unit volume (μg/μL), the number of flowers per plant, and the honey potential proposed by [92]. Furthermore, considering the average canopy width of *P. rotundum* var. *subintegrum* and the typical planting density of herbaceous species, the potential plant population per hectare was estimated at 110,000 plants/ha, allowing for conversion into honey production per hectare (kg/ha).

Honey yield (kg/ha) = Honey production (g/plant) ^a^ × Plant density or population (ea/ha) × 0.001 (for unit conversion: g to kg) × Honey potential ^b^

^a^ Honey production (g/tree) = Nectar volume (μL/flower) × Free sugar content (μg/μL) × Number of flower (ea/plant) × 0.00001 (for unit conversion: μg to g).

^b^ Honey potential = Sugar content:Honey = 85:100 [92].

### 4.10. The Data Analysis

Statistical analyses were performed using SPSS Statistics 19.0 (SPSS Inc., Chicago, IL, USA). A one-way analysis of variance (ANOVA) was conducted to examine variations in the growth characteristics, inflorescence morphology, number of inflorescences, number of flowers per inflorescence, photosynthetic response, chlorophyll content, chlorophyll fluorescence parameters, nectar secretion, amino acid composition, and potential honey production across the climate change scenarios. Levene’s test was applied to verify the homogeneity of variance assumptions, and Tukey’s HSD test was used for post hoc comparisons at a 5% significance level. Additionally, a repeated-measures ANOVA was performed to assess the variations in the free sugar content.

## 5. Conclusions

Climate change, characterized by rising atmospheric CO₂ concentrations and increasing temperatures, profoundly influences the growth, flowering phenology, nectar production, and chemical composition of nectariferous plants. These changes not only affect pollinator survival but also present substantial challenges to sustainable apiculture. *P. rotundum* var. *subintegrum* has been recognized for its high nectar yield and strong ecological adaptability, and this study further confirmed its resilience under projected climate change scenarios.

With an increasing climate change intensity, *P. rotundum* var. *subintegrum* exhibited enhanced net photosynthesis due to CO_2_ saturation in the intercellular spaces of the mesophyll tissues (Ci) before and after flowering, while maintaining a high photosynthetic efficiency, as indicated by its stable PI_ABS_ and SFI_ABS_ levels. To mitigate the effects of strong solar radiation and elevated temperatures, the plant displayed adaptive physiological responses, including a gradual reduction in photosynthetic pigments and an increase in its water-use efficiency. These adaptations facilitated sustained carbohydrate assimilation, which in turn promoted vegetative growth, increased inflorescence numbers, and enhanced the overall flower production. As a result, the total nectar yield per plant and per hectare increased up to 3.8-fold, despite the nectar secretion per flower remaining relatively stable.

Flowering onset and peak bloom also advanced under climate change scenarios, and accelerated carbohydrate hydrolysis contributed to the maintenance of nectar sugar homeostasis. However, while the overall nectar volume increased, a reduction in its sucrose content under the higher-emission scenarios (SSP3 and SSP5) resulted in a modest reduction in nectar quality, as indicated by a shift toward a sucrose-rich classification (0.5–1.0). The decrease in the amino acid content under the elevated CO_2_ and temperature conditions suggests that the nitrogen availability in the soil may have been depleted. This decline is likely due to the increased nitrogen demand for aboveground biomass accumulation, which could have limited the allocation of nitrogen to nectar production. Although a total of 17 amino acids were consistently detected across all scenarios, reductions in their overall concentrations—particularly in nitrogen-rich amino acids—suggest a potential decline in the nectar’s nutritional quality for pollinators. Despite this, phenylalanine, the most preferred amino acid by honeybees, consistently exhibited the highest proportion, indicating that *P. rotundum* var. *subintegrum* remained an important nectar source.

In summary, *P. rotundum* var. *subintegrum* exhibits strong climatic resilience by maintaining robust photosynthetic activity, increasing its floral abundance, and sustaining its nectar production under elevated temperature and CO_2_ conditions. While slight reductions in nectar quality—particularly in its nitrogen content and amino acid concentrations—were observed, the substantial increase in the nectar’s volume suggests that this species holds significant potential for apicultural site expansion in future climate scenarios. However, climate change encompasses not only warming and elevated CO_2_ levels but also extreme environmental stresses such as prolonged droughts and erratic precipitation. Given the critical role of nitrogen in amino acid biosynthesis, future research should explore the interactions between soil nitrogen availability, plants’ nitrogen metabolism, and nectar composition to ensure the long-term sustainability of pollinator–plant interactions and apicultural practices.

## Figures and Tables

**Table 1:** Changes in chlorophyll (Chl) and carotenoid (Car) contents of *P. rotundum* var. *subintegrum* under three SSP scenarios at different periods.

Month	SSP Scenarios	Chlorophyll (mg·g^−1^)			Carotenoid (mg·g−1)	Chl a/b	Total Chl/Car
		a	b	a + b			
May	SSP1	12.55 ± 0.75 b	2.88 ± 0.19 ns	15.43 ± 0.83 b	3.74 ± 0.39 ns	4.37 ± 0.20 ns	4.12 ± 0.29 b
	SSP3	10.38 ± 0.29 a	2.54 ± 0.15	12.92 ± 0.43 a	3.71 ± 0.13	4.09 ± 0.11	3.50 ± 0.07 a
	SSP5	11.90 ± 0.55 ab	2.94 ± 0.13	14.84 ± 0.65 ab	3.49 ± 0.25	4.07 ± 0.18	4.28 ± 0.28 b
July	SSP1	17.88 ± 1.91 c	4.40 ± 0.49 c	22.28 ± 2.39 c	4.51 ± 0.52 c	4.07 ± 0.22 ns	4.96 ± 0.22 b
	SSP3	14.10 ± 1.40 b	3.43 ± 0.27 b	17.53 ± 1.66 b	3.74 ± 0.29 b	4.11 ± 0.23	4.69 ± 0.45 b
	SSP5	10.67 ± 0.24 a	2.55 ± 0.26 a	13.21 ± 0.49 a	3.09 ± 0.29 a	4.19 ± 0.30	4.28 ± 0.35 a

Means with different letters indicate significant differences based on Tukey’s HSD test (*p <* 0.05); ns = non-significant.

**Table 2:** Growth and flowering characteristics of *P. rotundum* var. *subintegrum* under three SSP scenarios.

Treatments	Dry Weight (g)			S/R Ratio (g g^−1^)	Inflorescence		Number of Inflorescences per Plant	Number of Flowers per Inflorescence
	Shoot	Root	Total		Length (cm)	Width (cm)		
SSP1	104.5 ± 3.3 ^a^	56.4 ± 5.1 ^ns^	160.9 ± 7.7 ^a^	1.9 ± 0.1 ^a^	20.2 ± 2.4 ^a^	1.6 ± 0.4 ^ns^	50.1 ± 30.3 ^a^	205.0 ± 63.2 ^a^
SSP3	125.6 ± 9.7 ^ab^	45.2 ± 3.3	170.8 ± 6.7 ^ab^	2.8 ± 0.4 ^b^	30.4 ± 5.3 ^b^	1.8 ± 0.2	72.2 ± 31.3 ^b^	264.1 ± 50.6 ^a^
SSP5	143.0 ± 10.4 ^b^	52.2 ± 5.0	195.2 ± 15.6 ^b^	2.7 ± 0.1 ^b^	25.8 ± 6.3 ^b^	1.5 ± 0.2	89.0 ± 47.4 ^b^	347.4 ± 86.8 ^b^

Means with different letters indicate significant differences based on Tukey’s HSD test (*p <* 0.05). ns = non-significant.

**Table 3:** Nectar volume of *P. rotundum* var. *subintegrum* under three SSP scenarios at different periods.

Flowering Time	Treatments	Nectar Volume per Flower (μL/Flower)		
		BBS	MBS	EBS
1 day	SSP1	0.15 ± 0.01 ^a^	0.13 ± 0.05 ^ns^	0.21 ± 0.04 ^b^
	SSP3	0.15 ± 0.04 ^a^	0.10 ± 0.03	0.14 ± 0.05 ^ab^
	SSP5	0.26 ± 0.02 ^b^	0.15 ±0.07	0.09 ± 0.01 ^a^
2 day	SSP1	0.37 ± 0.09 ^ns^	0.28 ± 0.08 ^ns^	0.41 ± 0.05 ^b^
	SSP3	0.38 ± 0.10	0.28 ± 0.02	0.27 ± 0.05 ^a^
	SSP5	0.33 ± 0.22	0.31 ± 0.05	0.24 ± 0.03 ^a^

Means with different letters indicate significant differences based on Tukey’s HSD test (*p* < 0.05); ns = non-significant. BBS = beginning-bloom stage, MBS = mid-bloom stage, EBS = end-bloom stage.

**Table 4:** The *p*-values for the free sugar content of *P. rotundum* var. *subintegrum* under three SSP scenarios across different periods according to the repeated-measures ANOVA.

Source	Day 1				Day 2			
	Sucrose	Glucose	Fructose	FSC	Sucrose	Glucose	Fructose	FSC
Flowering stage (FS)	<0.001	<0.001	<0.001	<0.001	<0.001	<0.001	<0.001	<0.001
Treatment (T)	<0.001	<0.001	<0.001	<0.001	<0.001	<0.001	<0.001	<0.001
FS × T	<0.001	<0.001	<0.001	<0.001	<0.001	<0.001	<0.001	<0.001

**Table 5:** Results of repeated-measures ANOVA for amino acid content of *P. rotundum* var. *subintegrum* under three SSP scenarios.

Flowering Time	Treatments	BBS	MBS	EBS	Flowering Stage (FS)	Treatment (T)	FS × T
1 day (mg/L)	ssp1	2406.1 ± 23.2	4430.3 ± 187.1	2383.2 ± 179.2	<0.001	<0.001	<0.001
	ssp3	4419.8 ± 83.1	3520.1 ± 128.9	2788.1 ± 129.0			
	ssp5	1585.9 ± 16.4	1476.6 ± 72.2	2513.4 ± 60.4			
2 day (mg/L)	ssp1	2373.6 ± 121.8	4335.0 ± 157.7	2693.2 ± 281.7	<0.001	<0.001	<0.001
	ssp3	1836.8 ± 37.9	3385.8 ± 178.8	2872.3 ± 184.0			
	ssp5	2257.3 ± 59.3	1198.5 ± 89.5	2437.6 ± 52.3			

BBS = beginning-bloom stage, MBS = mid-bloom stage, EBS = end-bloom stage.

**Table 6:** Estimation of honey production based on nectar secretion and flowering characteristics of *P. rotundum* var. *subintegrum* under three SSP scenarios.

Treatments	Nectar Sugar Content (μg/Flower)	Estimated Honey Production (g/Plant)	Estimated Honey Yield (kg/ha)
SSP1	0.22 ± 0.03 ^ns^	2.6 ± 0.3 ^a^	285.8 ± 36.1 ^a^
SSP3	0.17 ± 0.02	3.6 ± 0.5 ^a^	401.1 ± 56.3 ^a^
SSP5	0.20 ± 0.05	7.1 ± 1.6 ^b^	772.8 ± 178.8 ^b^

Means with different letters indicate significant differences based on Tukey’s HSD test (*p <* 0.05); ns = non-significant.

**Table 7:** Summary of key physiological and nectar traits of *P. rotundum* var. *subintegrum* across three SSP scenarios.

Trait Category	Indicator	SSP1	SSP3	SSP5
Physiological responses	Net photosynthetic rate	Lowest	Moderate in May; decreased in July	Highest (↑1.3 × SSP1)
	Intercellular CO_2_ concentration	Baseline	Increased	Highest
	Instantaneous transpiration efficiency	Lowest	↑2.2–2.4 × SSP1	
	PI_ABS_/SFI_ABS_	Increased from May to July	Moderate increase	Highest in May, lower in July
	Total chlorophyll contents	Highest	Decreased to 78.0–78.8% of that in SSP1	Decreased to 57.8–59.6% of the value in SSP1
Growth characteristics	Aboveground biomass	Baseline	+20.2%	+36.9%
	Number of inflorescences	50.1 ± 30.3	72.2 ± 31.3	89.0 ± 47.4
	Number of flowers per inflorescence	205.0 ± 63.2	264.1 ± 50.6	347.4 ± 86.8
	Flowering onset	28 June	26 June	21 June
Nectar quantity	Nectar volume per flower (EBS, day 2)	Highest	Moderate	59.9% lower than that in SSP1
	Total estimated honey per plant	2.6 g	3.6 g	7.1 g
	Estimated yield per ha	317.0 kg	401.1 kg	772.8 kg
Nectar quality	Sucrose content (EBS, day 2)	279.9 µg/µL	314.6 µg/µL	8.6% lower than that in the early stage
	Free sugar content	431.1–555.5 µg/µL	Variable	Peaked in the BBS, day 2
Amino acids	Total amino acid content (MBS, day 2)	Highest (4335.0 mg/L)	High	Lowest (1198.5 mg/L)
	Phenylalanine	62.5–75.5%		

**Table 8:** Summary of the chlorophyll fluorescence parameters from the OKJIP test.

Parameters	Description
V_K_/V_J_	The ratio of variable fluorescence in a time of 0.3 ms to the variable fluorescence in a time of 2 ms as an indicator of the PSII’s donor-side limitation
ABS/RC	Absorption flux per reaction center
TR_0_/RC	Trapped energy flux per reaction center at t = 0
ET_0_/RC	Electron transport flux from QA to QB per reaction center at t = 0
DI_0_/RC	Energy dissipation flux per reaction center at t = 0
RE_0_/RC	Electron transport flux until PSI acceptors per reaction center at t = 0
Φ_PO_	Probability that an absorbed photon leads to a reduction further than Q_A_^-^
Φ_EO_	Probability that an absorbed photon leads to electron transport further than Q_A_^-^
Ψ_O_	Probability that an absorbed photon leads to a reduction in Q_A_^-^
PI_ABS_	The performance index on an absorption basis
SFI_ABS_	The structure function index on an absorption basis

## Data Availability

The raw data supporting the conclusions of this article will be made available by the authors upon request.
